# loneliness, solitude and social desintegration in the elderly and their relationship to health

**DOI:** 10.1192/j.eurpsy.2023.126

**Published:** 2023-07-19

**Authors:** G. Stoppe

**Affiliations:** University of Basel, MentAge, Basel, Switzerland

## Abstract

**Abstract:**

Loneliness has become a big issue in the time of the COVID pandemic. The attention to the topic also has to do with the increase in people living alone in Europe, although this also has to do with prosperity. Living alone does not yet mean being lonely. In the scientific discussion and especially in the measures, a differentiation must be made between loneliness, solitude and social disintegration. Poor social integration is easily measurable and has a lot to do with the physical health (mobility, vision and hearing) of the people concerned. However, the extent to which participation in social and cultural opportunities is possible, for example through the expansion of public transport, also plays an important role.

Loneliness, on the other hand, is by definition subjective and strongly linked to mental health. It describes the subjective suffering of missing or unsatisfactory social relationships, lack of integration and security. Loneliness is often found in two peaks, among the young and the old. Political and public health campaigns often focus on social integration measures. However, measures to combat loneliness mean first and foremost recognising mental illness in old age, especially depression. But they also mean providing help to people with long-term mental illness in old age.

**Disclosure of Interest:**

None Declared

